# Risk factors assessment and a Bayesian network model for predicting ischemic stroke in patients with cardiac myxoma

**DOI:** 10.3389/fcvm.2023.1128022

**Published:** 2023-03-24

**Authors:** Lin Ma, Bin Cai, Man-Li Qiao, Ze-Xin Fan, Li-Bo Fang, Chao-Bin Wang, Guang-Zhi Liu

**Affiliations:** ^1^ Department of Neurology, Beijing Anzhen Hospital, Capital Medical University, Beijing, China; ^2^ Department of General Practice Medicine, Beijing Anzhen Hospital, Capital Medical University, Beijing, China; ^3^ Department of Neurology, Beijing Fangshan District Liangxiang Hospital, Beijing, China; ^4^ Department of Neurology, Beijing Fuxing Hospital, Capital Medical University, Beijing, China

**Keywords:** Bayesian network, ischemic stroke, cardiac myxoma, prediction model, risk factor

## Abstract

**Objective:**

This study aims to identify relevant risk factors, assess the interactions between variables, and establish a predictive model for ischemic stroke (IS) in patients with cardiac myxoma (CM) using the Bayesian network (BN) approach.

**Methods:**

Data of patients with CM were collected from three tertiary comprehensive hospitals in Beijing from January 2002 to January 2022. Age, sex, medical history, and information related to CM were extracted from the electronic medical record system. The BN model was constructed using the tabu search algorithm, and the conditional probability of each node was calculated using the maximum likelihood estimation method. The probability of each node of the network and the interrelationship between IS and its related factors were qualitatively and quantitatively analyzed. A receiver operating characteristic (ROC) curve was also plotted. Sensitivity, specificity, and area under the curve (AUC) values were calculated and compared between the BN and logistic regression models to evaluate the efficiency of the predictive model.

**Results:**

A total of 416 patients with CM were enrolled in this study, including 61 with and 355 without IS. The BN model found that cardiac symptoms, systemic embolic symptoms, platelet counts, and tumor with high mobility were directly associated with the occurrence of IS in patients with CM. The BN model for predicting CM-IS achieved higher scores on AUC {0.706 [95% confidence interval (CI), 0.639–0.773]} vs. [0.697 (95% CI, 0.629–0.766)] and sensitivity (99.44% vs. 98.87%), but lower scores on accuracies (85.82% vs. 86.06%) and specificity (6.56% vs. 11.48%) than the logistic regression model.

**Conclusion:**

Cardiac symptoms, systemic embolic symptoms, platelet counts, and tumor with high mobility are candidate predictors of IS in patients with CM. The BN model was superior or at least non-inferior to the traditional logistic regression model, and hence is potentially useful for early IS detection, diagnosis, and prevention in clinical practice.

## Introduction

Ischemic stroke (IS) is a common and severe complication of cardiac myxoma (CM) (1–3). Despite a low incidence of 5 per 10,000,000 individuals per year (4), CM is generally considered an important etiology of stroke in young adult (5–7), and nearly half of the patients who suffer CM-related ischemic stroke (CM-IS) have multiple cerebral infarcts on brain imaging (8, 9). The high morbidity and fatality of CM-IS places a great burden on one's family and society (10). Hence, early identification of IS in patients with CM is important as it can improve clinical outcomes and reduce medical costs. Thus far, the risk factors of CM-IS remain largely uncertain. Although several risk factors of embolism (i.e., size of tumor and sex) (11, 12) in CM-IS patients have been reported, they are still controversial. Currently, the occurrence of CM-IS is believed to result from the interaction of multiple risk factors such as age (13–15), sex (12, 16–18), past medical history (19, 20), and tumor characteristics (21–23).

In general, traditional analysis methods such as logistic regression have poor statistical effects when managing collinear/high-dimensional data. In contrast, a Bayesian network (BN) can accurately reflect the potential relationship and the strength of the relationship between variables by constructing a directed acyclic graph and conditional probability table. Additionally, mounting evidence has proven the successful application of BN models in medical diagnosis, expert systems, statistical decision-making, learning, and prediction. Given the unavailability of an agreed set of guidelines or reports on the development of prediction models for IS in CM cohorts, there is a great need to establish highly predictive models for early IS detection and diagnosis. In this study, we identified relevant risk factors, assessed the interactions between variables, established a predictive model for IS in patients with CM using the BN approach, and further compared the model performance between traditional logistic regression and BN models.

## Materials and methods

### Study population and clinical data collection

We retrospectively reviewed 468 patients who were diagnosed with CM and underwent cardiac surgery in three tertiary comprehensive hospitals between January 2010 and January 2022. The following inclusion criteria were adopted: (i) patients diagnosed with CM by echocardiography before surgery; (ii) patients with postoperative pathological reports confirmed as CM; and (iii) patients with single CM or presenting for the first time with CM. The exclusion criteria were as follows: (i) patients with valvular heart disease, congenital heart disease, deep venous thrombosis, history of infective endocarditis, cardiac thrombosis, other tumors, migraine, unstable carotid artery plaques, or patients taking oral contraceptives; (ii) patients with missing clinical data. When a patient was admitted more than twice, only the data from the first admission were used in this study. The study was performed in accordance with the Declaration of Helsinki and approved by the Institutional Ethics Committee of Beijing Anzhen Hospital (2023013X). Written informed consent was obtained from all participants.

Demographic and clinical data were collected from electronic medical records, including age, sex, medical history, clinical symptoms, echocardiography, and laboratory tests (i.e., routine blood examination and coagulation function). Patients with IS were diagnosed based on medical history, clinical examination, and brain imaging results before cardiac surgery, and their diagnoses were confirmed by two attending neurologists. The collected variables during admission: including clinical, imaging, and laboratory data, were used in the statistical analysis and BN modeling.

With regard to echocardiographic data, CM with high-mobility refers to highly mobile phenomena of myxoma mass on transesophageal echocardiography as described elsewhere (1, 24). Additionally, we chose 30 mm as the threshold of the size of CM, because two previous studies reported that tumor diameter less than 30 mm were more commonly seen in patients with CM complicated by brain infarct (25).

After hospital discharge, follow-ups were performed by experienced investigators *via* face-to-face interviews or phone calls. The main outcomes included complications and mortality, which were monitored for periods lasting 1 month to 10 years.

### Quality control

The data extraction process from the medical records was standardized, and the investigators familiarized themselves with it before starting the data retrieval for this study. Data entry followed the double entry method. If discrepancies take place during the review process, medical records were consulted and the data were corrected.

### Data processing for predictive variables

Before building the predictive model, we reviewed the previous literature to preprocess the predictive variables. First, it was noted that CM-IS mainly affects middle-aged women (20, 26). For our analysis, sex and age were defined as the definite risk factors. Second, in addition to the traditional cerebrovascular risk factors [such as smoking (27, 28), hypertension (29), diabetes (30), and hyperlipidemia (31)], we also added “tumor characteristics” as risk factors involving tumor length, width, mobility, surface and stalk (11, 32). Furthermore, previous studies have demonstrated the clinical manifestations of CM, including cardiac, constitutional, and systemic embolic symptoms (4, 33). Elevated platelet counts have also been reported in CM-IS (12). Based on the above-mentioned findings, a total of 17 variables were identified, including sex, age, hypertension, diabetes, hyperlipidemia, tumor type, tumor size, tumor location, cardiac symptoms, constitutional symptoms, systemic embolic symptoms, and platelet count.

According to the biostatistics literature, data will lose their measure of confidence if their missing value ratio is >30%. These missing attributes normally result from time conflicts and test failures. In our study, some variables were removed from the dataset if they had more than six missing attributes. Finally, approximately six variables were used as the primary dataset.

### Bayesian network

A BN consists of a directed acyclic graph whose nodes represent random variables and links express dependencies between nodes (34). The node indicated by an arrow is called the child node, and the other node is called the parent node of the child node. Each node corresponds to a conditional probability distribution table (CPT), which represents the conditional probability of the child node under the condition that the parent node is known and quantifies the probability dependence between variables. The BN is a representation of the joint probability distributions of random variables *X* = [*X*_1_, …, *X_n_*]; thus, a probability expression can be obtained:

$$P(X_{1},\ldots ,X_{n})=P(X_{1})P(X_{2}|X_{1})\ldots P(X_{n}|X_{1},X_{2},\ldots X_{n-1})=\prod_{1}^{n}P(X_{i}|\pi (X_{i})$$

Where *π*(*X_i_*) represents the collection of the parents of *X*i; *π*(*X_i_*) ⊆ *X*_1_ …, *X_i_*_-1_.

The purpose of BN structure learning is to find the network that best matches a given dataset. Tabu Search (TS) is currently the most used search method for structure learning as it is able to overcome issues related to the local optimal searching process and can search for the global optimal solution. For the network structure G and the training dataset D, parameter learning modifies the parameters of the prior distribution and obtains the parameter of the posterior distribution, which determines the BN of the conditional probability of each node to *p*-value *(D | θ, G)*. Maximum likelihood estimation is one of the main types of estimation for a complete dataset, and this method simply involves an attempt to find the parameter that maximizes the likelihood function. In this study, we constructed a BN model to predict CM-IS occurrence. 17 random variables were extracted from the patient data. We initially filtered the nodes using logistic regression to avoid including too many nodes and adding excessive complexity to the network structure. We established an optimal model based on the tabu search algorithm and maximum-likelihood estimation.

### Statistical analysis

Continuous variables are presented as mean ± standard deviation or median [interquartile range (IQR)]. Categorical variables are expressed as percentages. Normally distributed data were analyzed using Student's *t*-test, and non-normally distributed data were analyzed using the Mann–Whitney *U* test. Categorical variables were analyzed using the chi-square test. Binary logistic regression analysis was used to screen for possible variables associated with CM-IS and evaluate their associated risk intensities. Variables with *p*-value < 0.05 were entered into the multivariable logistic regression analysis to identify the risk factors.

The BN model of the IS-related risk factors in patients with CM was conducted using the tabu search algorithm (35), and the conditional probability of each node was calculated using the maximum likelihood estimation method (36). The probability of each node of the network and the interrelationship between IS and related factors were qualitatively and quantitatively analyzed (37). Before establishing the BN model, all the IS-related factors were quantified and coded (Supplementary Table S1). The performance of the BN model was evaluated through discrimination and calibration. The discriminative ability of the model was reflected by the area under the receiver operating characteristic curve (AUC). Calibration was performed using the Hosmer–Lemeshow test and calibration plots. Furthermore, the Delong test was used to test the statistical significance of the difference between the AUC values. The BN model for predicting the probability of CM-IS was constructed using the bnlearn package in R 4.2.1 (https://www.rstudio.com/). In addition, the network topology was drawn using Netica 32.0 (Norsys Software Corp., Vancouver, BC, Canada). The AUC was used to evaluate the prediction efficiency of the model, which was completed using R studio software (https://www.rstudio.com/).

## Results

### Demographic and clinical characteristics

**Figure 1:**
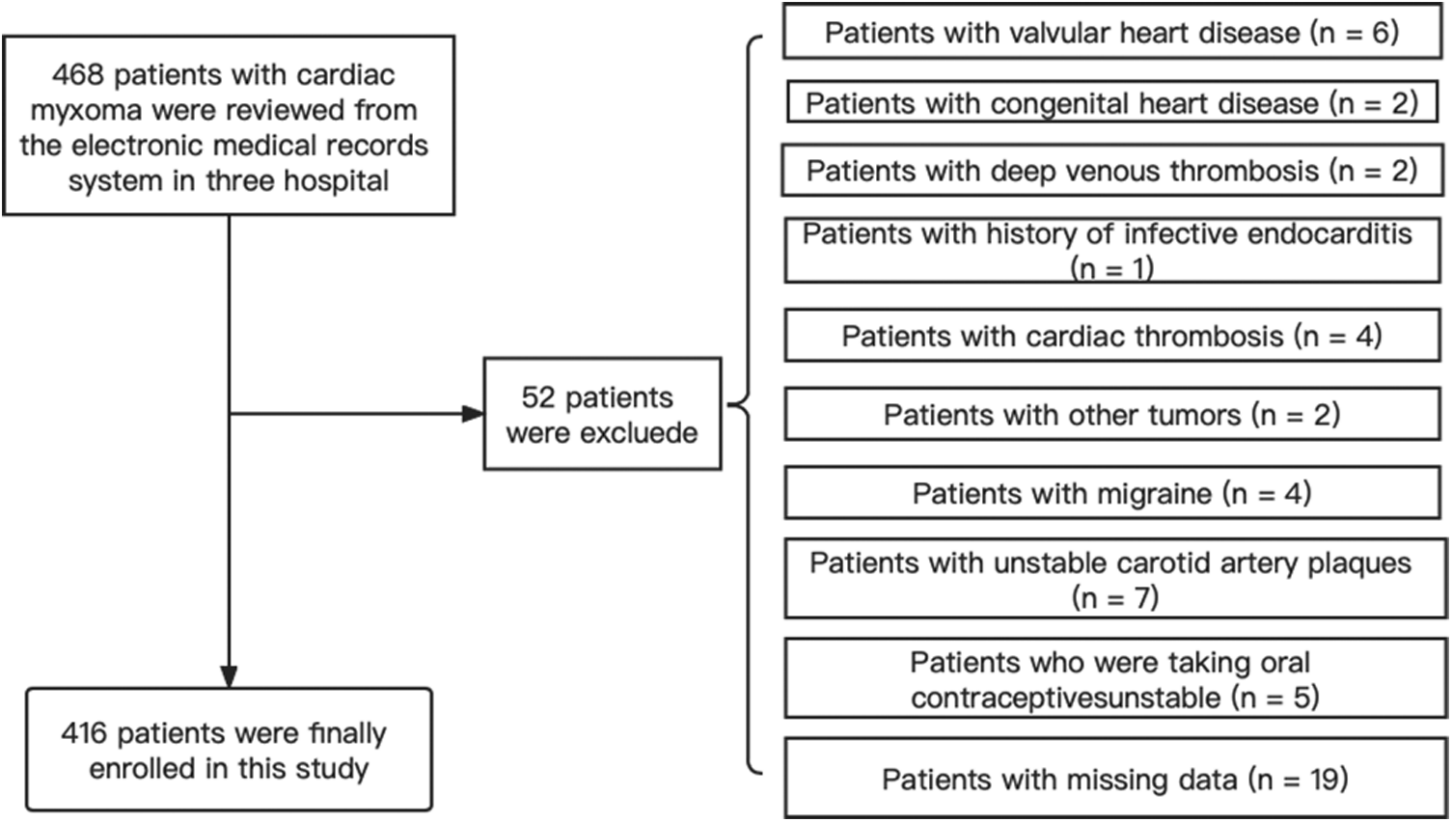
Flowchart describing the screening of patients with cardiac myxoma (CM).

Among 468 consecutive patients with CM, 52 were excluded due to the presence of other concomitant diseases or missing data. Finally, 416 patients were enrolled in this study, including 61 with IS and 355 without IS (Figure 1). Baseline characteristics were also compared between the CM-IS (61 patients) and non-IS CM (355 patients) groups. The mean age of the participants included in this study was 53.81 years, and 62.0% of them were women. Compared with non-IS CM group (*n* = 355), patients with CM-IS more frequently had hypertension (42.6% vs. 28.2%, *p*-value = 0.023), hyperlipidemia (19.7% vs. 7.0%, *p*-value = 0.001), clinical manifestations of cardiac symptoms (65.5% vs. 58.9%, *p*-value < 0.001), and systemic embolic symptoms (9.8% vs. 1.1%, *p*-value < 0.001). Approximately 42 (68.6%) patients with CM-IS had tumors with high mobility (*p*-value = 0.03). The additional patient characteristics are presented in Table 1.

**Table 1:** Baseline characteristics of patients with cardiac myxoma.

Variables	Total (*n* = 416)	CM-IS (*n* = 61)	CM-non IS (*n* = 355)	*p*-value
Age, years	53.81 ± 13.87	56.17 ± 13.10	53.41 ± 13.97	0.151
Female, *n* (%)	258 (62.0)	30 (49.2)	228 (64.2)	0.081
**Medical history, *n* (%)**				
Hypertension	126 (30.3)	26 (42.6)	100 (28.2)	0.023
Hyperlipidemia	37 (8.9)	12 (19.7)	25 (7.0)	0.001
Diabetes mellitus	47 (11.3)	8 (13.1)	39 (11.0)	0.628
**Smoking status, *n* (%)**				0.355
Non-smoker	300 (72.1)	39 (63.8)	262 (73.8)	
Current smoker	84 (20.2)	15 (24.6)	69 (19.4)	
Ex-smoker	32 (7.7)	7 (11.4)	24 (7.0)	
**Clinical feature, *n* (%)**				
Cardiac symptoms	229 (55.0)	40 (65.5)	209 (58.9)	<0.001
Constitutional symptoms	9 (2.2)	0 (0)	9 (2.5)	1
Systemic embolic symptoms	10 (2.4)	6 (9.8)	4 (1.1)	<0.001
Platelets counts (10^9^/L)	228.25 ± 83.44	253.35 ± 89.09	223.94 ± 81.79	0.011
**Echocardiography**				
Length, mm	36.41 ± 20.60	36.59 ± 20.79	36.38 ± 20.59	0.940
Width, mm	25.16 ± 15.47	22.78 ± 14.29	25.57 ± 15.62	0.193
High mobility, *n* (%)	149 (35.8)	42 (68.6)	107 (30.1)	0.03
Irregular surface, *n* (%)	1 (0.2)	0 (0)	1 (0.3)	1
Stalk, *n* (%)	301 (72.4)	47 (77.0)	254 (71.5)	0.375
LVEF, %	61.17 ± 10.43	61.64 ± 9.52	60.09 ± 10.59	0.704

CM-IS, cardiac myxoma-related ischemic stroke; CM-non IS, cardiac myxoma without ischemic stroke; LVEF, left ventricular ejection fractions.

Regarding the outcomes of patients with CM after surgical excision, two patients had a stroke recurrence (two CM-IS cases) and three patients died (one CM-IS case, two non-IS CM cases). Causes of death were acute exacerbation of chronic heart failure (*n* = 1), acute myocardial infarction (*n* = 1), and severe lung infection (*n* = 1).

### Risk factors in the CM-IS model

The variables included in this study were the basic characteristics and well-known risk factors. The results of the univariate logistic regression analysis are listed in Table 2. Of the 17 variables, six were associated with CM-IS according to the univariate logistic regression: hyperlipidemia [odds ratio (OR), 1.426; 95% confidence interval (CI), 1.187–1.970; *p*-value = 0.042], hypertension (OR, 1.537; 95% CI, 1.291–1.990; *p*-value = 0.046), cardiac symptoms (OR, 2.772; 95% CI, 2.515–5.071; *p*-value = 0.001), systemic embolic symptoms (OR, 1.172; 95% CI, 1.045–1.662; *p*-value < 0.010), platelet count (OR, 1.005; 95% CI, 1.001–1.008; *p*-value = 0.006), and tumors with high mobility (OR, 2.493; 95% CI, 1.661–5.643; *p*-value = 0.013).

**Table 2:** Risk factors of CM-IS: univariate and multivariate binary logistic regression analysis.

Characteristics	Univariate analysis		Multivariate analysis	
	OR (95% CI)	*p*-value	OR (95% CI)	*p*-value
Hyperlipidemia				
No	1.00 (Reference)	-	1.00 (Reference)	-
**Yes**	**1.426 (1.187–1.970)**	**0.042**	1.172 (0.940–5.019)	0.69
Hypertension				
No	1.00 (Reference)	-	1.00 (Reference)	-
**Yes**	**1.537 (1.291–1.990)**	**0.046**	1.937 (1.044–3.595)	0.36
Cardiac symptom				
No	1.00 (Reference)	-	1.00 (Reference)	-
**Yes**	**2.772 (2.515–5.071)**	**0.001**	**2.363 (1.198–0.667)**	**0.001**
Systemic embolic symptoms				
No	1.00 (Reference)	-	1.00 (Reference)	-
**Yes**	**1.172 (1.045–1.662)**	**0.010**	**1.324 (0.683–20.495)**	**0.015**
**Platelets counts (10^9^/L)**	**1.005 (1.001–1.008)**	**0.006**	**1.006 (1.001–1.008)**	**0.008**
Tumor mobility				
Low	1.00 (Reference)	-	1.00 (Reference)	-
**High**	**2.493 (1.661–5.643)**	**0.013**	**2.579 (1.292–5.148)**	**0.017**

CM-IS, cardiac myxoma-related ischemic stroke; OR, odds ratio; CI, confidence interval.

In our research, six independent variables were included in the multivariate analysis. As the sample size should be at least 10 times the independent variable when developing a prediction model for dichotomous outcomes, the number of samples in each group should be at least 60. The number of cases of CM-IS and non-IS CM were 61 and 355, respectively. Thus, the sample size of this study was sufficient to develop a predictive model. Therefore, six significant variables were retained in the final multivariate logistic regression model after performing backward stepwise variable selection. In the multivariable analysis, hyperlipidemia (OR, 1.172; 95% CI, 0.940–5.019; *p*-value = 0.69), hypertension (OR, 1.937; 95% CI, 1.044–3.595; *p*-value = 0.36), cardiac symptoms (OR, 2.363; 95% CI, 1.198–0.667; *p*-value = 0.001), systemic embolic symptoms (OR, 1.324; 95% CI, 0.683–20.495; *p*-value = 0.015), platelet count (OR, 1.006; 95% CI, 1.001–1.008; *p*-value = 0.008), and tumors with high mobility (OR, 2.579; 95% CI, 1.292–5.148; *p*-value = 0.017) were independently associated with CM-IS (Table 2).

### Structure and parameters of the BN

**Figure 2:**
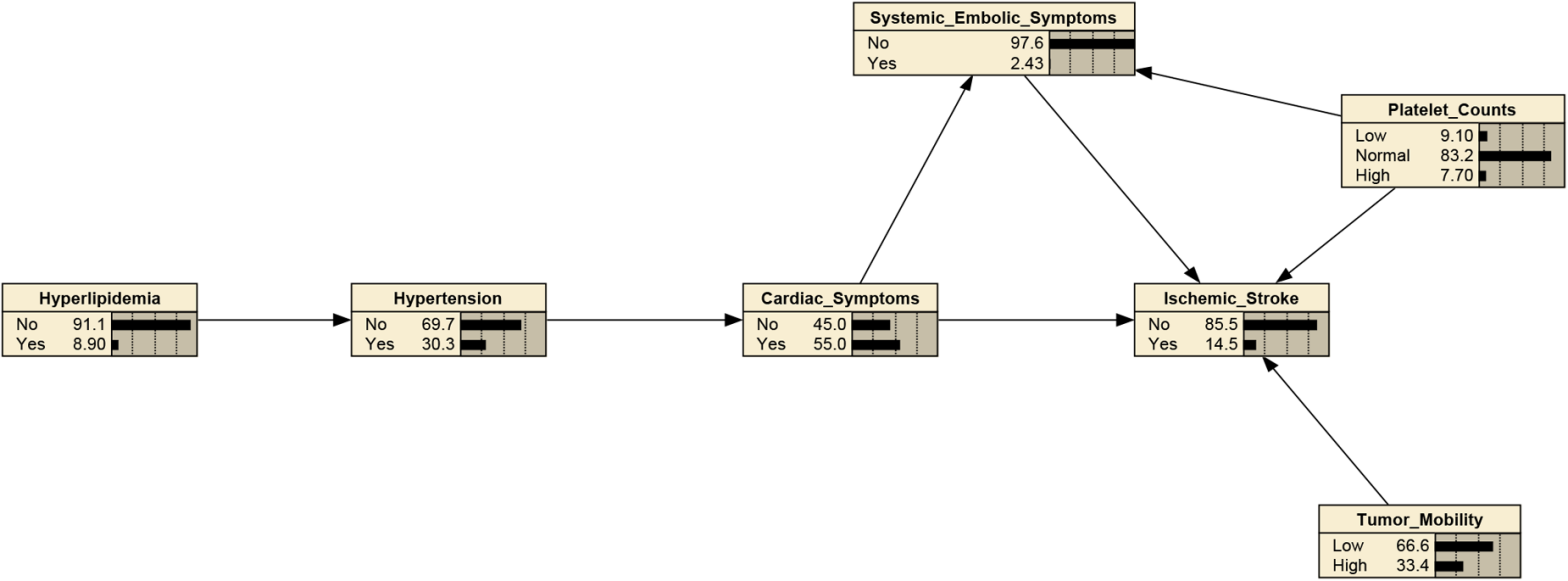
Bayesian network (BN) for predicting occurrence of IS in patients with cardiac myxoma (CM). The Bayesian network used 6 variables selected by mutivariate binary logistic regression.

The structure learning of the BN was mainly constructed using the tabu search algorithm. The CM-IS model contained seven nodes and 10 directed edges, with nodes representing hypertension, hyperlipidemia, cardiac symptoms, systemic embolic symptoms, platelet counts, and tumor mobility, respectively. As shown in Figure 2, hypertension, hyperlipidemia, cardiac symptoms, systemic embolic symptoms, platelet counts, and tumor with high mobility were directly associated with CM-IS, and tumor with high mobility was indirectly linked to CM-IS through cardiac symptoms and systemic embolic symptoms. Hypertension may also be indirectly associated with CM-IS *via* cardiac and systemic embolic symptoms.

After determining the BN structure, the maximum likelihood estimation method was used to estimate the conditional probabilities of each node in the network. The prior probabilities of each node in the network are shown in Figure 2. According to the conditional probability distribution (Table 3), when CM was combined with systemic embolic symptoms and abnormal platelet counts, the probability of CM-IS was 0.50. In patients with high platelet counts complicated by cardiac symptoms and with co-occurring systemic embolic symptom disturbance, the probability of IS increased to 1.00, suggesting that great attention should be paid to prevent the occurrence of CM-IS when the above two phenomena occur.

**Table 3:** The conditional probability distributions with tumors mobility, platelets counts, cardiac and systemic embolic symptoms as parent nodes.

Cardiac symptoms	Systemic embolic symptoms	Platelets counts	Tumor mobility	Ischemic stroke (%)	
				Yes	No
No	No	Low	Low	0.25	0.75
No	No	Low	High	1.00	0.00
Yes	No	Low	Low	1.00	0.00
Yes	No	Low	High	1.00	0.00
No	Yes	Low	Low	0.00	1.00
No	No	Normal	Low	0.22	0.78
No	No	Normal	High	0.16	0.84
Yes	No	Normal	Low	0.10	0.90
Yes	No	Normal	High	0.09	0.91
No	Yes	Normal	Low	0.50	0.50
No	Yes	Normal	High	1.00	0.00
No	No	High	Low	0.43	0.57
No	No	High	High	0.00	1.00
Yes	No	High	Low	1.00	0.00
Yes	Yes	High	Low	1.00	0.00

### Model performance evaluation

**Figure 3:**
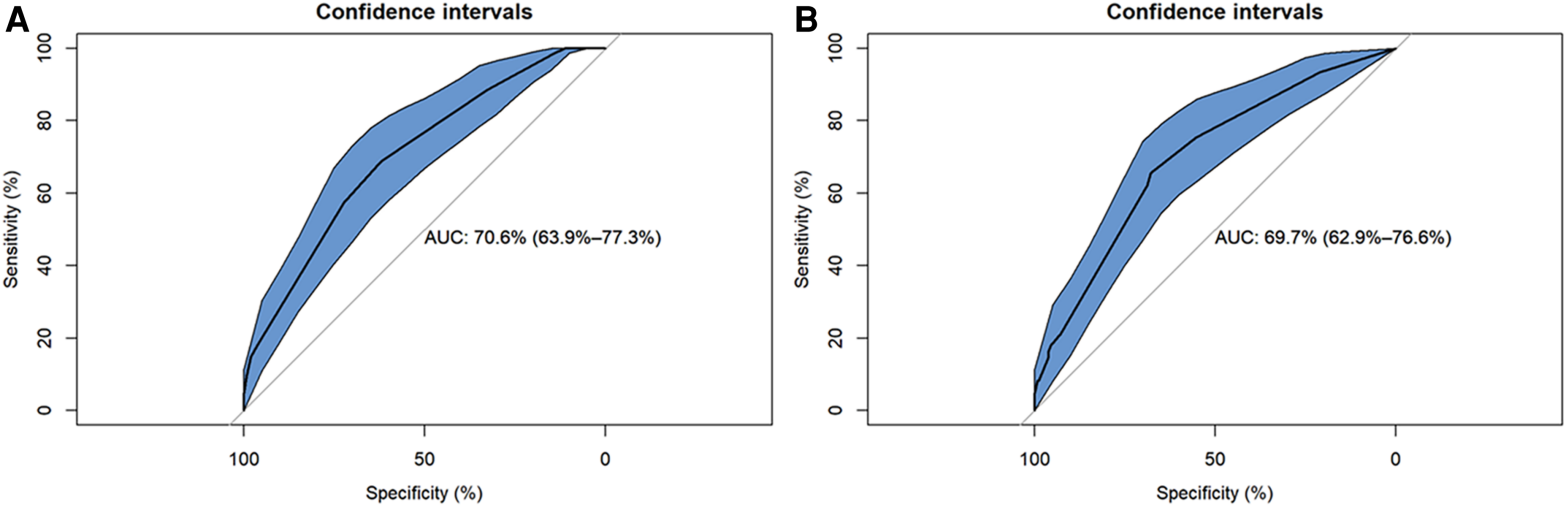
Reciver-Operationg Characteristic (ROC) curves of Bayesian network (BN) model and logistic regression model for predicting ischemic stroke (IS) in patients with cardiac myxoma (CM). (**A**) Logistic regression model. (**B**) Bayesian network model.

Table 4 shows that the BN model for predicting CM-IS achieved higher scores on AUC and sensitivity, but lower scores on accuracies and specificity, than the logistic regression model. The AUC of the BN model was 0.706 (95% CI, 0.639–0.773), and its accuracies, sensitivity, and specificity were 85.82%, 99.44%, and 6.557%, respectively. The logistic regression predictive model achieved an AUC of 0.697 (95% CI, 0.629–0.766), accuracies of 86.06%, sensitivity of 98.87%, and specificity of 11.48% (Figure 3).

**Table 4:** The performance of different predictive models.

Model	Accuracy	AUC	Sensitivity	Specificity
Bayesian network	85.82%	0.706	99.44%	6.56%
Logistic regression	86.06%	0.697	98.87%	11.48%

AUC, area under the curve; CI, confidence interval.

**Figure 4:**
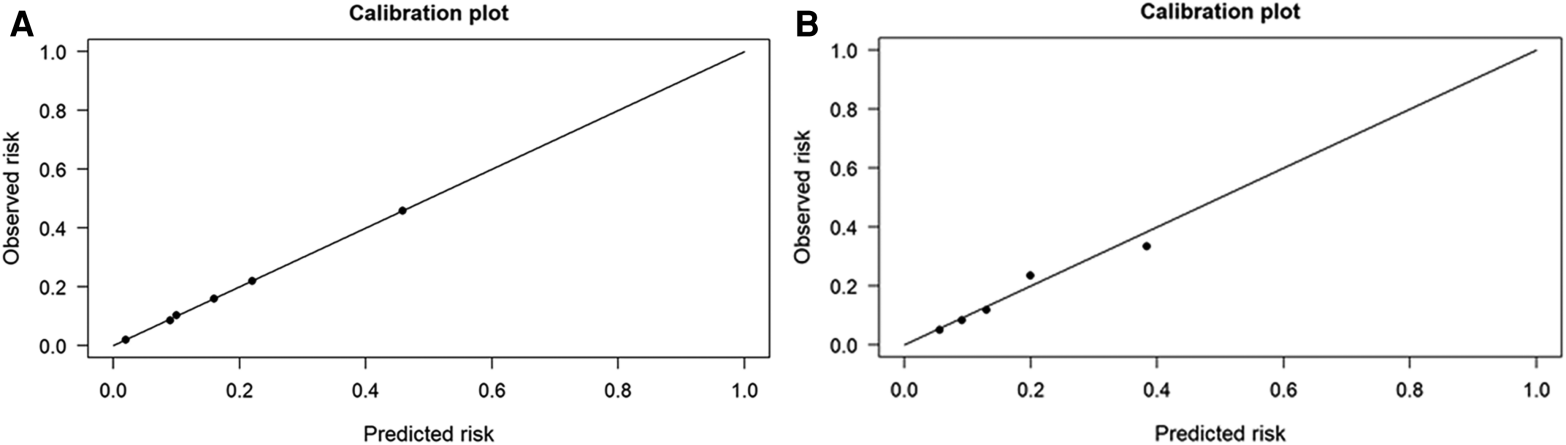
The performances of Bayesian network (BN) model and logistic regression model for predicting ischemic stroke (IS) in patients with cardiac myxoma (CM). (**A**) Logistic regression model. (**B**) Bayesian network model.

Notably, the Delong test revealed that there were no statistical differences in the AUC values between the BN model and the logistic regression model (*p*-value = 0.683). In addition, the calibration plots showed that the predicted probabilities of IS agreed well with the actual observations (Figure 4), and the Hosmer–Lemeshow test also demonstrated good calibration for the BN model (*p*-value = 1.000, *χ*^2^ = 0.033, degrees of freedom = 8) and for the logistic regression model (*p*-value = 0.992, *χ*^2^ = 1.536, degrees of freedom = 8).

## Discussion

In the present study, univariate and multivariate logistic regression models were used to identify the main risk factors of IS in patients with CM. Next, a BN model was constructed to estimate the conditional probability of each node based on a univariate analysis using the tabu search algorithm. Our study found that cardiac symptoms, systemic embolic symptoms, tumor with high mobility, and elevated platelet counts were directly associated with the occurrence of CM-IS. Hypertension is indirectly linked to IS through systemic embolic symptoms. In addition, the BN model achieved better AUC and sensitivity than the traditional logistic regression model.

CM mainly affects young female subjects, as reported in previous studies. Consistent with this, our results also showed that approximately 62% of the enrolled patients were female. However, our study showed no obvious female predominance in patients with CM-IS, with a female-to-male ratio of 1:1. A potential explanation for this result might be the exclusion of patients taking oral contraceptives and patients with migraine in our study to reduce interference from other causes of stroke. In addition, the mean patient age was consistent with that of the CM population. Lee et al. reported that stroke patients with CM had an average age of 48.5 (range, 17–70) years (17). Furthermore, a case series by Ekinci et al. revealed patients with CM were aged between 6 and 82 years. The marked relationship between age and CM-IS in our study was in agreement with previous studies, strongly suggesting that CM should be highly suspected in young patients with IS.

In our study, traditional cerebrovascular risk factors such as hypertension, hyperlipidemia, and smoking were directly linked to CM-IS, implying that such risk factors, which are common among patients with CM-IS, still need to be strictly monitored and managed to prevent the occurrence of CM-IS. Moreover, in a series of 113 atrial myxoma patients with neurological presentations, 83% presented with IS (12). Thus, some clinical manifestations may be related to CM-IS as risk factors, including systemic embolic, cardiac, and/or constitutional symptoms (39). Indeed, our results revealed that the incidence of cardiac symptoms in patients with CM-IS (65.5%) was relatively low compared to that in patients without IS (58.9%), and hypertension was indirectly associated with IS through systemic embolic symptoms using the BN model, further substantiating our viewpoint.

Previous studies have also found that tumor characteristics (i.e., tumor length, tumor width, tumor mobility, tumor surface, and tumor stalk) might be potential predictors of embolism in patients with left atrial tumors (21, 22, 32). According to an embolism event risk scoring system based on these tumor characteristics, patients with scores ≥ 7 (7/11) had a 45% risk for embolic events and those with scores ≥ 9 had a 75% risk (11). In our study, tumors with high mobility were directly associated with IS. This finding is noteworthy because it indicates that the morphology of the tumor rather than the size of the tumor may predispose patients to IS occurrence. Nevertheless, further investigation is needed to determine the relationship between CM morphology and IS.

In addition to the above, a retrospective cohort study by He et al. reported that higher platelet count (>300 × 10^9^/L) and mean platelet volume were associated with IS in patients with CM. However, the association between platelet count and CM remains controversial (12, 40). Regarding the underlying mechanism, we can only speculate that elevated platelets may increase blood viscosity and aggravate red blood cell adhesion, which consequently contributes to embolism events (41).

A BN can further intuitively describe the interaction between independent variables and the complex network between independent and dependent variables while also analyzing the influencing factors, which is helpful for comprehensively exploring the causal associations among diseases and discovering unknown potential risk factors (37). In practice, a BN reflects the potential relationship and strength between multiple factors using directed acyclic graphs and conditional probability distribution tables (36, 38). In previous studies, the risk prediction model of CM-IS was independently established based on multivariate logistic regression (19, 20, 42). However, this cannot satisfy the need to distinguish between direct risk factors and mediating factors, thus failing to infer the interaction of each influencing factor. In the BN model, hypertension was also indirectly associated with IS through cardiac symptoms, indicating that the BN might be more suitable for sequential clinical processes. Moreover, the BN provides a visualized probability of CM-IS. In addition, a BN possesses a certain adaptability regarding processing data with strong robustness against missing values.

The incidence rate of CM is approximately 5 per 10,000,000 (4), and CM accounts for less than 1% of embolic stroke (43). Previous research on CM-IS mainly consisted of case reports and small cohort studies (19). Under such circumstances, conclusions are often incomprehensive, with relatively limited evidence. BN models are suitable for processing limited domain knowledge, which is then simplified or extended by inputting new knowledge to meet various requirements. Clinicians can supplement patients’ up-to-date data, enabling the BN model to automatically adjust the probabilities. For instance, if the patient's platelet counts >300 × 10^9^/L, with cardiac symptoms, systemic embolic symptom, but without high tumor mobility, the probability was 1.0. However, this doesn't mean that IS is certain to occur under such circumstances, but only indicates that such patients with CM have a probability of 1.0 for concurrent IS. Therefore, our results suggested that the BN model based on the tabu search algorithm exhibited a flexible inference mechanism that helps detect and diagnose IS in patients with CM and, more importantly, helps prevent the occurrence and recurrence of IS.

A BN is a powerful machine learning method for classifying imbalanced datasets. In fact, real-world datasets are often challenged by class imbalances. Our study also had unbalanced classes in the IS prediction (355: 61). With regard to AUC and sensitivity, the BN model performed relatively better when compared to the traditional logistic regression model, although the difference was not statistically significant. In clinical practice, the factors utilized for model prediction may be missing, leading to the model being unable to make predictions. However, the BN had better radial basis and multilayer perceptron sensitivity. Thus, in the present study, the learning process searched for the best BN structure and for parameters and as a result, demonstrated the higher AUC (>0.7) and sensitivity than the traditional logistic regression model. The BN model in this study was able to deal with missing data and predict IS risks in patients with CM based on limited information (e.g., with only the subjects’ basic characteristics and results from simple accessory tests), rather than special data (e.g., brain neuroimaging data). Hence, such a dataset for developing a predictive model for IS can be easily utilized in community clinics or rural hospitals.

## Limitation of the study

The present study had some limitations. First, considering the low incidence rates of CM and CM-IS, the number of confirmed cases with CM was small, particularly for CM-IS. Second, regarding the number of events, the study failed to split the data into training and test sets, which may have influenced the accuracy of the predictive BN model. Thirdly, considering the low mortality and morbility in patients with CM, it is difficult to estimate the association between IS and clinical outcomes. Further research is needed to determine this. Finally, selection bias may exist owing to the disparity in patient enrollment between the three medical centers.

## Conclusion

In summary, we first proposed a BN model to predict IS in patients with CM, achieving a better or at least non-inferior performance than the traditional logistic regression model. Cardiac symptoms, systemic embolic symptoms, platelet counts, and tumors with high mobility were candidate predictors of IS in our patient cohort. Hence, the model is potentially helpful for early IS detection, diagnosis, and disease prevention in clinical practice. Nevertheless, larger multicenter, prospective studies are still needed to ascertain the causal relationship between risk factors and CM-IS, optimize treatment strategies, in order to improve the disease prognosis of patients with CM.

## Data Availability

The raw data supporting the conclusions of this article will be made available by the authors, without undue reservation.

## References

[1] Lee VH Connolly HM Brown RD. Central nervous system manifestations of cardiac myxoma. Arch Neurol. (2007) 64:1115–20. 10.1001/archneur.64.8.1115 17698701

[2] Yuan SM Humuruola G. Stroke of a cardiac myxoma origin. Rev Bras Cir Cardiovasc. (2015) 30:225–34. 10.5935/1678-9741.20150022 26107455 PMC4462969

[3] Zhang Y Ye Z Fu Y Zhang Z Ye Q Chen F Characterizations of ischemic stroke complications in cardiac myxoma patients at a single institution in eastern China. Neuropsychiatr Dis Treat. (2021) 17:33–40. 10.2147/NDT.S280641 33442253 PMC7800702

[4] Keeling IM Oberwalder P Anelli-Monti M Schuchlenz H Demel U Tilz GP Cardiac myxomas: 24 years of experience in 49 patients. Eur J Cardiothorac Surg. (2002) 22:971–7. 10.1016/S1010-7940(02)00592-4 12467822

[5] Namaganda P Nakibuuka J Kaddumukasa M Katabira E. Stroke in young adults, stroke types and risk factors: a case control study. BMC Neurol. (2022) 22:335. 10.1186/s12883-022-02853-5 36068544 PMC9446773

[6] Bejot Y Delpont B Giroud M. Rising stroke incidence in young adults: more epidemiological evidence, more questions to be answered. J Am Heart Assoc. (2016) 5:e003661. 10.1161/JAHA.116.003661 27169549 PMC4889213

[7] Ekker MS Verhoeven JI Vaartjes I van Nieuwenhuizen KM Klijn CJ de Leeuw FE. Stroke incidence in young adults according to age, subtype, sex, and time trends. Neurology. (2019) 92:e2444–54. 10.1212/WNL.0000000000007533 31019103

[8] Domanski O Dubois R Jegou B. Ischemic stroke due to a cardiac myxoma. Pediatr Neurol. (2016) 65:94–5. 10.1016/j.pediatrneurol.2016.08.014 27743744

[9] Kacar P Pavsic N Bervar M Straza ZD Zadnik V Jelenc M Cardiac myxoma: single tertiary centre experience. Radiol Oncol. (2022) 56(4):535–40. 10.2478/raon-2022-0041 36259335 PMC9784375

[10] Jiang CX Wang JG Qi RD Wang W Gao LJ Zhao JH Long-term outcome of patients with atrial myxoma after surgical intervention: analysis of 403 cases. J Geriatr Cardiol. (2019) 16:338–43. 10.11909/j.issn.1671-5411.2019.04.003 31105754 PMC6503479

[11] Elbardissi AW Dearani JA Daly RC Mullany CJ Orszulak TA Puga FJ Embolic potential of cardiac tumors and outcome after resection: a case-control study. Stroke. (2009) 40:156–62. 10.1161/STROKEAHA.108.525709 18948602

[12] He DK Zhang YF Liang Y Ye SX Wang C Kang B Risk factors for embolism in cardiac myxoma: a retrospective analysis. Med Sci Monit. (2015) 21:1146–54. 10.12659/MSM.893855 25900256 PMC4418206

[13] Abu AM Saleh S Alhaddad E Alsmady M Alshehabat M Bani IZ Cardiac myxoma: clinical characteristics, surgical intervention, intra-operative challenges and outcome. Perfusion. (2017) 32:686–90. 10.1177/0267659117722596 28762298

[14] Karabinis A Samanidis G Khoury M Stavridis G Perreas K. Clinical presentation and treatment of cardiac myxoma in 153 patients. Medicine. (2018) 97:e12397. 10.1097/MD.0000000000012397 30213011 PMC6155961

[15] Coffee E Sankhla N Bass R Dure L Rashid S. Child neurology: arterial ischemic stroke in a 12-year-old patient with cardiac myxomas. Neurology. (2020) 94:e1103–6. 10.1212/WNL.0000000000009060 32071165

[16] Pinede L Duhaut P Loire R. Clinical presentation of left atrial cardiac myxoma. A series of 112 consecutive cases. Medicine. (2001) 80:159–72. 10.1097/00005792-200105000-00002 11388092

[17] Lee SJ Kim JH Na CY Oh SS. Eleven years’ experience with Korean cardiac myxoma patients: focus on embolic complications. Cerebrovasc Dis. (2012) 33:471–9. 10.1159/000335830 22517375

[18] Forbes LM Hensley ND Miller YE. A 58-year-old woman with a history of cardiac myxoma presents with pulmonary nodules. Chest. (2021) 160:e351–5. 10.1016/j.chest.2021.04.060 34625183

[19] Stefanou MI Rath D Stadler V Richter H Hennersdorf F Lausberg HF Cardiac myxoma and cerebrovascular events: a retrospective cohort study. Front Neurol. (2018) 9:823. 10.3389/fneur.2018.00823 30337904 PMC6178925

[20] Yuan L Ge L Zhu Y Chen C Zhou Z Yang Q. Cardiac myxoma and ischemic stroke. QJM. (2020) 113:674–5. 10.1093/qjmed/hcaa085 32142144

[21] Gosev I Paic F Duric Z Gosev M Ivcevic S Jakus FB Biocina B. Cardiac myxoma the great imitators: comprehensive histopathological and molecular approach. Int J Cardiol. (2013) 164:7–20. 10.1016/j.ijcard.2011.12.052 22243936

[22] Oliveira R Branco L Galrinho A Abreu A Abreu J Fiarresga A Cardiac myxoma: a 13-year experience in echocardiographic diagnosis. Rev Port Cardiol. (2010) 29:1087–100. 10.1016/S0300-8932(10)70197-4 21066964

[23] Wang H Li Q Xue M Zhao P Cui J. Cardiac myxoma: a rare case series of 3 patients and a literature review. J Ultrasound Med. (2017) 36:2361–6. 10.1002/jum.14264 28556391

[24] Ha JW Kang WC Chung N Chang BC Rim SJ Kwon JW Echocardiographic and morphologic characteristics of left atrial myxoma and their relation to systemic embolism. Am J Cardiol. (1999) 83(11):1579–82. 10.1016/S0002-9149(99)00156-3 10363879

[25] Cao GF Bi Q Cao L Wang C. The clinical characteristics of stroke in young patients with cardiac myxoma. Zhonghua Nei Ke Za Zhi. (2017) 56(4):263–7. 10.3760/cma.j.issn.0578-1426.2017.04.005 28355718

[26] Gaszewska-Zurek E Zurek P Wilczynski M Krzych L Bachowski R Jasinski M Cardiac myxoma - clinical presentation and long-term post-operative follow-up. Kardiol Pol. (2011) 69:329–34. 10.1002/clc.20844 21523664

[27] Larsson SC Burgess S Michaelsson K. Smoking and stroke: a mendelian randomization study. Ann Neurol. (2019) 86:468–71. 10.1002/ana.25534 31237718 PMC6701987

[28] Markidan J Cole JW Cronin CA Merino JG Phipps MS Wozniak MA Smoking and risk of ischemic stroke in young men. Stroke. (2018) 49:1276–8. 10.1161/STROKEAHA.117.018859 29674522 PMC5916531

[29] Cipolla MJ Liebeskind DS Chan SL. The importance of comorbidities in ischemic stroke: impact of hypertension on the cerebral circulation. J Cereb Blood Flow Metab. (2018) 38:2129–49. 10.1177/0271678X18800589 30198826 PMC6282213

[30] Lau LH Lew J Borschmann K Thijs V Ekinci EI. Prevalence of diabetes and its effects on stroke outcomes: a meta-analysis and literature review. J Diabetes Investig. (2019) 10:780–92. 10.1111/jdi.12932 30220102 PMC6497593

[31] Rawshani A Rawshani A Franzen S Sattar N Eliasson B Svensson AM Risk factors, mortality, and cardiovascular outcomes in patients with type 2 diabetes. N Engl J Med. (2018) 379:633–44. 10.1056/NEJMoa1800256 30110583

[32] Kalcik M Bayam E Guner A Kup A Kalkan S Yesin M Evaluation of the potential predictors of embolism in patients with left atrial myxoma. Echocardiography. (2019) 36:837–43. 10.1111/echo.14331 30934139

[33] Liao WH Ramkalawan D Liu JL Shi W Zee CS Yang XS The imaging features of neurologic complications of left atrial myxomas. Eur J Radiol. (2015) 84:933–9. 10.1016/j.ejrad.2015.02.005 25737060

[34] Zhang Z Zhang J Wei Z Ren H Song W Pan J Application of tabu search-based Bayesian networks in exploring related factors of liver cirrhosis complicated with hepatic encephalopathy and disease identification. Sci Rep. (2019) 9:6251. 10.1038/s41598-019-42791-w 31000773 PMC6472503

[35] Liu Z Malone B Yuan C. Empirical evaluation of scoring functions for Bayesian network model selection. BMC Bioinform. (2012) 13(Suppl 15):S14. 10.1186/1471-2105-13-S15-S14 PMC3439716 23046392

[36] Zhang X Yuan Z Ji J Li H Xue F. Network or regression-based methods for disease discrimination: a comparison study. BMC Med Res Methodol. (2016) 16:100. 10.1186/s12874-016-0207-2 27538955 PMC4991108

[37] Jin Z Kang J Yu T. Feature selection and classification over the network with missing node observations. Stat Med. (2022) 41:1242–62. 10.1002/sim.9267 34816464 PMC9773124

[38] Brownstein NC Cai J. Tests of trend between disease outcomes and ordinal covariates discretized from underlying continuous variables: simulation studies and applications to NHANES 2007-2008. BMC Med Res Methodol. (2019) 19:2. 10.1186/s12874-018-0630-7 30611216 PMC6321711

[39] Wen XY Chen YM Yu LL Wang SR Zheng HB Chen ZB Neurological manifestations of atrial myxoma: a retrospective analysis. Oncol Lett. (2018) 16:4635–9. 10.3892/ol.2018.9218 30214598 PMC6126161

[40] Liu Y Wang J Guo L Ping L. Risk factors of embolism for the cardiac myxoma patients: a systematic review and metanalysis. BMC Cardiovasc Disord. (2020) 20:348. 10.1186/s12872-020-01631-w 32711463 PMC7382866

[41] Dias RR Fernandes F Ramires FJ Mady C Albuquerque CP Jatene FB. Mortality and embolic potential of cardiac tumors. Arq Bras Cardiol. (2014) 103:13–8. 10.5935/abc.20140096 25029470 PMC4126756

[42] Galvez-Ruiz A Galindo-Ferreiro A Lehner AJ Kozac I. Clinical presentation of multiple cerebral emboli and central retinal artery occlusion (CRAO) as signs of cardiac myxoma. Saudi J Ophthalmol. (2018) 32:151–5. 10.1016/j.sjopt.2017.09.001 29942186 PMC6010594

[43] Hart RG Diener HC Coutts SB Easton JD Granger CB O'Donnell MJ Embolic strokes of undetermined source: the case for a new clinical construct. Lancet Neurol. (2014) 13:429–38. 10.1016/S1474-4422(13)70310-7 24646875

