# Supplementary material for: Risk factors assessment and a Bayesian network model for predicting ischemic stroke in patients with cardiac myxoma
Source: Front Cardiovasc Med. 2023 Mar 24;10:1128022. doi: 10.3389/fcvm.2023.1128022 (PMC10079949; doi:10.3389/fcvm.2023.1128022)
Supplement: Supplementary file 1 [file Table1.pdf]

Supplementary Material

Risk factors assessment and a Bayesian network model for predicting ischemic stroke in patients with cardiac myxoma

Lin Ma[1†], Bin Cai[1†], Man-Li Qiao[2†], Ze-Xin Fan[1], Li-Bo Fang[3], Chao-Bin Wang[4], Guang-Zhi Liu[1*]

*** Correspondence:** Guang-Zhi Liu, guangzhi2002@hotmail.com

1 Supplementary Data

None

2 Supplementary Figures and Tables

2.1 Supplementary Figures

None

2.2 Supplementary Tables

Table S1. Risk factors and assignment of ischemic stroke (IS) in cardiac myxoma (CM)

Variables	Assignment
Hypertension	no = 0, yes = 1
Hyperlipidemia	no = 0, yes = 1
Cardiac symptoms	no = 0, yes = 1
Systemic embolic symptoms	no = 0, yes = 1
Platelets counts (10^9/L)	≤100 = 0, 100-300 = 1, ≥300 = 2
Tumor mobility	low = 0, high = 1
Ischemic stroke	no = 0, yes = 1
